# Trauma plays an important role in acral melanoma: A retrospective study of 303 patients

**DOI:** 10.1002/cam4.7137

**Published:** 2024-03-28

**Authors:** Rong Huang, Mengke Zhao, Guiying Zhang, Yueling Yang, Jiayu Wang, Kelin Zheng, Lin Li, Xinyu Su, Lianjun Zhao, Yirong Wu, Zhengyun Zou

**Affiliations:** ^1^ Department of the Comprehensive Cancer Center, Nanjing Drum Tower Hospital, Affiliated Hospital of Medical School Nanjing University Nanjing China; ^2^ Nanjing Drum Tower Hospital Clinical College of Nanjing Medical University Nanjing China; ^3^ Nanjing Drum Tower Hospital Clinical College of Nanjing University of Chinese Medicine Nanjing China

**Keywords:** acral melanoma, clinical classification, clinicopathologic characteristics, genetic landscape, trauma, tumor immune microenvironment

## Abstract

**Introduction:**

Acral melanoma (AM) is the most common subtype of malignant melanoma in China, with a very poor prognosis. Despite the frequent reporting of trauma events in AM cases, the precise etiology of AM remains elusive.

**Methods:**

A retrospective analysis was conducted on a cohort of 303 AM patients at Nanjing Drum Tower Hospital. The patients were categorized into four distinct groups based on different patterns of disease onset: trauma type (Type 1), pigmented nevus type (Type 2), pigmented nevi with trauma (Type 3), and pigmented nevi with natural ulceration (Type 4). Differences in clinicopathological features, genetic alterations, and tumor immune microenvironment (TIME) were analyzed.

**Results:**

Traumatic events accounted for a large proportion of AM cases. Among these categories, Type 1 patients displayed the least favorable pathological traits and an immunosuppressive TIME. Common copy number variations (CNVs) were observed in CCND1, RB1, FGF19, and IL7R, while CNVs in CDK4 and TERT occurred less frequently in patients with a history of trauma (Type 1 and Type 3). Type 2 patients exhibited the most favorable pathological characteristics and genetic profiles, and demonstrated the lowest incidence of CCDN1 and RB1 CNVs but had the highest CDK4 CNVs. In contrast, the pathological behavior of Type 3 and Type 4 patients was in between Type 1 and Type 2. And patients in Type 3 and Type 4 displayed a more favorable overall microenvironment.

**Conclusion:**

This study provides a clinical classification of Chinese AM based on diverse clinical onset characteristics and highlights the important role of trauma in AM. These findings may help to guide the diagnosis, treatment, and prognosis of AM patients. Further investigations are imperative to elucidate the underlying mechanisms governing the association between trauma and AM.

## Graphical abstract

Our manuscript aims to investigate the role of trauma in the onset of Chinese acral melanoma (AM) and propose a novel clinical classification system. Trauma‐onset AM, primarily featured the histological subtype of nodular melanoma, advanced T stages, the highest NRAS mutation rate, and the highest M2 macrophage infiltration in the invasive margin. Therefore, our study underscores the important role of trauma in AM.

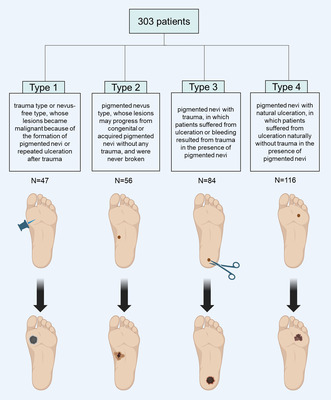

## 1 INTRODUCTION

Melanoma is a kind of highly malignant cancer that has witnessed a global surge in incidence over the past few decades, making it an increasingly pressing public health concern. Between 1990 and 2019, the number of newly diagnosed cases of malignant melanoma in the world increased by 170%, from 107,380 to 289,950.^1^ In China, the age‐standardized incidence rate of melanoma increased by 0.6% every year from 1990 to 2005, and by 6.1% every year from 2005 to 2017.^2^ Different from Caucasians, whose melanomas are mostly located at the non‐acral skin and related to chronic sun damage, melanoma cases in Chinese population mainly occur on the extremities, precisely the hands and feet, considered not to be directly linked to sun exposure.^3^, ^4^

The etiology of acral melanoma (AM) remains an enigma,^5^ and the current mainstream view is related to genetic alterations and trauma.^6^ For instance, BRAF and NRAS mutations play an important role in cutaneous melanoma, especially in those related to sun damage.^6^ In contrast, AM cases more commonly exhibit mutations and amplifications of the KIT gene and amplifications of the CCND1 gene.^6^, ^7^ Epidemiologically, trauma has been linked to an increased risk of melanoma at the trauma site and the incidence of traumatic events in AM has been reported to be approximately 20.8%–26.8%.^5^, ^8^, ^9^ Some scholars posit that trauma may facilitate melanoma formation by augmenting DNA mutations and the release of related cytokines.^10^ Nevertheless, relevant studies are limited and the available evidence remains inconclusive.

This study aims to investigate the role of trauma in the onset of Chinese AM and propose a novel clinical classification system. We have categorized AM patients into four distinct types based on their diverse onset characteristics and subsequently conducted a comprehensive analysis of disparities in clinicopathological features, genetic changes, and tumor immune microenvironment (TIME) across these four categories.

## 2 METHODS

### 2.1 Patient collection

A total of 303 AM patients were collected consecutively from July 2010 to October 2021 at affiliated Drum Tower Hospital, Medical School of Nanjing University. All patients included in this study had been diagnosed as AM by histopathology. Detailed clinicopathological data were recorded, including the name, gender, age, primary tumor site, onset pattern, histological subtype, and TNM stage, which was defined according to the eighth AJCC TNM staging system. Clinical onset patterns were subsequently verified through clinical records and telephone follow‐up. Melanoma patients with their sites on the dorsal acral were excluded in this study due to the possibility to sun exposure and their genetic alterations closer to non‐acral cutaneous melanoma.^11^ This study was approved by the Ethics Committee of the Affiliated Drum Tower Hospital, Medical School of Nanjing University (approval no. 2022‐170‐02).

### 2.2 Genetic testing and multiplexed immunohistochemistry

A total of 105 patient samples were subjected to targeted next‐generation sequencing (NGS) by Nanjing Geneseeq Technology Inc. before receiving their treatment. High‐throughput sequencing was performed by the PE150 kit of Illumina Hiseq sequencing platform, with an average sequencing depth of not less than 1000X. Tumor mutation burden (TMB) was calculated as the number of non‐synonymous somatic mutations, including missense, nonsense, splice‐site, frameshift, and inframe mutations. Among them, 76 patient samples received multiplexed immunohistochemistry (mIHC) detection for TIME. Five immune cell subsets in the TIME were identified by mIHC and multispectral imaging. The CD8 marker was used to identify T cells, while CD56 was used to identify natural killer (NK) cells and divide them into two categories according to the intensity of membrane staining for CD56 protein: CD56dim (weak staining) and CD56bright (strong staining). Based on CD68 and HLA‐DR, macrophages were classified into two categories: subtype M1 (CD68^+^ and HLA‐DR^+^) and subtype M2 (CD68^+^ and HLA‐DR^−^). The invasive margin and tumor center were determined using S100 staining. The positive rate, defined as the ratio of the number of positive cells and the total number of cells in the tumor center or invasive margin, was performed to assess the degree of immune infiltration.

### 2.3 Statistical analysis

Statistical analyses were performed using SPSS version 26.0. Constituent ratios and associations between covariates were assessed using chi‐squared tests and Fisher's exact tests. Differences were evaluated through Kruskal–Wallis tests and Mann–Whitney test. Figures were generated using GraphPad Prism (version 8.4.0) and the R project (version 4.3.3). Univariate survival data were generated utilizing the Kaplan–Meier method, complemented by log‐rank tests. The overall survival metric was calculated from the time of the initial diagnosis to the last follow‐up or date of death. Statistical significance was defined as *p* ≤ 0.05.

## 3 RESULTS

### 3.1 Clinical characteristics

**FIGURE 1:**
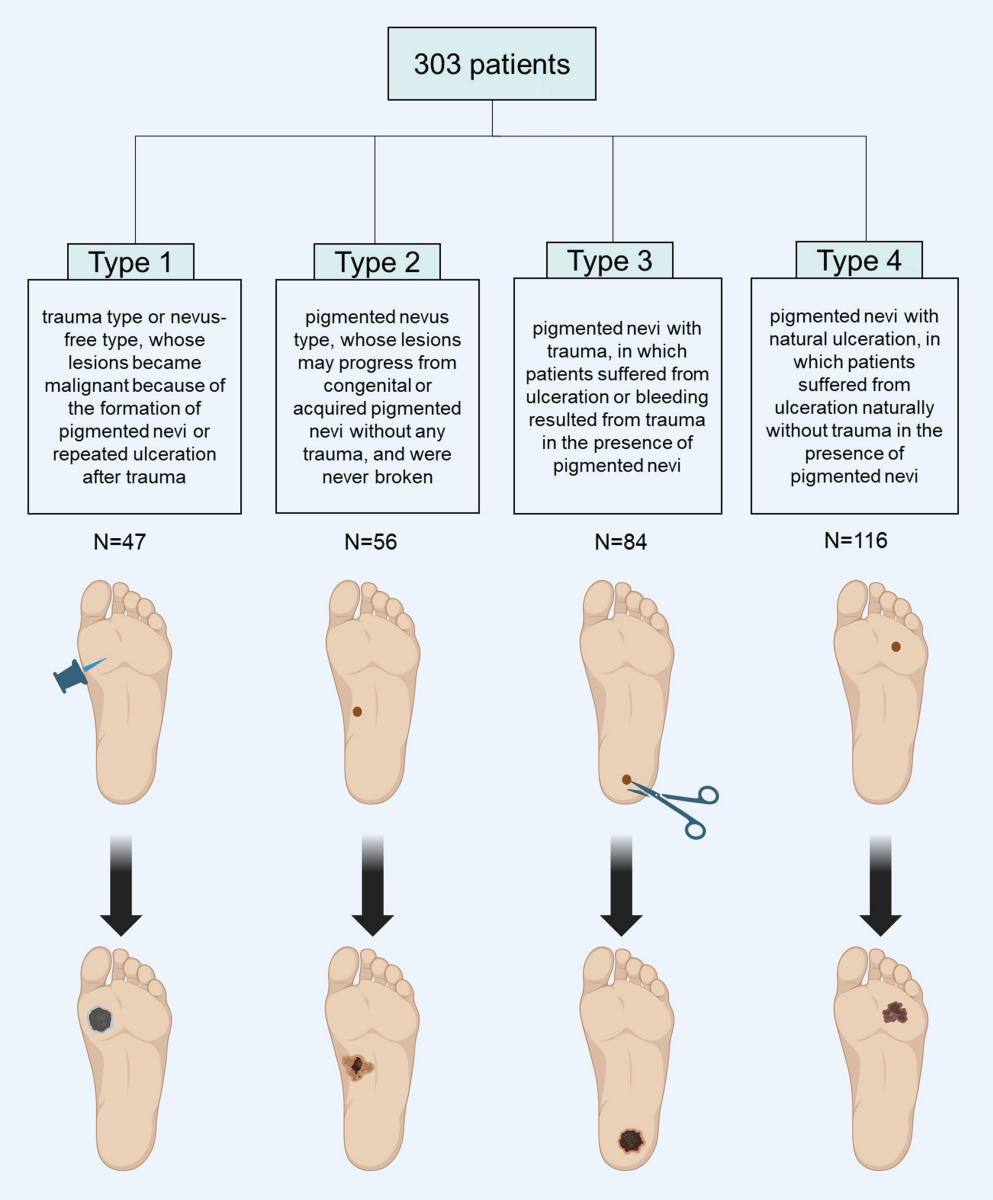
A new clinical classification of acral melanoma according to the different onset characteristics. In total, 303 acral melanoma patients were divided into four types according to their different onset characteristics: Type 1, trauma type; Type 2, pigmented nevus type; Type 3, pigmented nevi with trauma; Type 4, pigmented nevi with natural ulceration. (This figure was created with BioRender, https://biorender.com/).

Based on different onset characteristics, our cohort of 303 patients was categorized into four distinct types; Trauma type (Type 1): Patients in this category exhibited lesions that transformed from nevus‐free areas to malignancy, following repeated ulceration due to all kinds of traumas commonly encountered in agricultural work. These kinds were described in detail including stab wounds (caused by iron nails, straw, and wood chips), incised wounds (caused by nail clippers, agricultural cutting tools, and pointed stones), bruises or crushes by heavy objects, scald wounds by molten iron, medical intervention, and abrading. Pigmented nevus type (Type 2): These patients had lesions that may have originated from congenital or acquired pigmented nevi without any trauma, and these nevi had not experienced any damage or rupture. Pigmented nevus with trauma (Type 3): This group consisted of patients who experienced ulceration or bleeding as a result of trauma occurring in preexisting pigmented nevi. Pigmented nevus with natural ulceration (Type 4): Patients in this category developed ulceration naturally without any traumatic incidents while having pigmented nevi. Our study population comprised 47, 56, 84, and 116 patients, respectively, across these four types (shown in Figure 1).

The gender distribution among the four types of patients was fairly balanced, with more males than females. The median age at initial diagnosis was 60 years old. Notably, Type 2 had a higher proportion of younger patients, while Type 4 exhibited the opposite trend. Across all four types, more than half of the patients had a lower level of education, referring to not exceeding 9‐year compulsory education including illiteracy, primary school education, and junior high school education. Notably, patients with a trauma‐related onset had the lowest level of education, with at least 80.9% of them not having received a senior high school education. However, there was no significant difference in educational attainment among the four groups (Pearson chi‐squared 5.311, *p* = 0.150). A summary of the clinical and histopathological characteristics of the 303 patients can be found in Table 1.

**TABLE 1:** The clinical and pathological characteristics.

Characteristic	Type 1	Type 2	Type 3	Type 4	Total
Numbers	47	56	84	116	303
Gender					
Male	31	25	48	62	166
Female	16	31	36	54	137
Age					
<60	25	34	41	52	152
≥60	22	22	43	64	151
T					
Tis	0	9	0	2	11
T1	3	12	3	4	22
T2	4	12	8	26	50
T3	13	7	18	29	67
T4	14	9	36	36	95
Unknown	13	7	19	19	58
N					
Positive	16	13	28	31	88
Negative	22	39	43	72	176
Unknown	9	4	13	13	39
M					
M0	47	54	74	109	284
M1	0	2	10	7	19
Ulceration					
Present	23	11	45	72	151
Absent	8	27	17	19	71
Unknown	16	18	22	25	81
Ki67					
<30%	12	17	16	24	69
≥30%	15	5	22	32	74
Unknown	20	34	46	60	160
AJCC stage					
0	0	9	0	2	11
I	4	16	4	9	33
II	14	14	36	56	120
III	17	13	26	32	88
IV	0	2	10	7	19
Unknown	12	2	8	10	32
Histological subtype					
In situ	0	9	0	2	11
NM	10	4	16	19	49
ALM	4	18	14	21	57
SSM	2	3	5	6	16
Others^a^	1	1	1	0	3
Undescribed	30	21	48	68	167
Education degree					
Illiteracy	11	8	14	30	63
Primary school	11	12	17	26	66
Junior high school	16	14	20	25	75
Senior high school	4	12	15	22	53
University/college	3	5	11	10	29
Unknown	2	5	7	3	17
Lesion sites					
Plantar	15	28	42	55	140
Heel	7	2	21	25	55
Subungual	8	15	8	13	44
Toes	10	7	9	17	43
Fingers	6	4	4	6	20

*Note*: The lesion site of only one patient in Type 1 was located on the palm. Others were summarized to be located on the five sites.

Abbreviations: ALM, acral lentiginous melanoma; M, distant metastasis; N, regional lymph node metastasis; NM, nodular melanoma; SSM, superficial spreading melanoma; T, T stage.

^a^ The others include malignant freckle‐like melanoma, spindle cell melanoma, and fibroproliferative melanoma.

We also collected the information on the duration of lesion presence, including the time from traumatic event to the diagnosis of melanoma. A total of 289 patients recalled the duration of lesion presence and these disease courses were non‐normally distributed with a median of 2.00 years and interquartile range of 4.00 years. However, there was no significant difference in the course of disease among the four groups (Kruskal–Wallis test, *p* = 0.311), as shown in Table S1; Figure S1.

### 3.2 Distribution of lesion sites

The anatomical sites of the lesions in our cohort of 303 patients were predominantly on the lower limbs (257, 85%) and upper limbs (46, 15%). Among the lower limb lesions, they were further subdivided into subungual (19, 7.4%), plantar (140, 54.5%), heel (55, 21.4%), and toes (43, 16.7%). The upper limb lesions included subungual (25, 55%), palm (1, 2%), and fingers (20, 43%). The distribution of lesion sites was shown in Table S2 in the Supplement.

Differences in the composition of lesion sites were observed among the four onset types. As shown in Table 1, Type 1 lesions predominantly occurred on the plantar region, the site of predilection for limb trauma. Moreover, roughly half of Type 2, Type 3, and Type 4 lesions also presented on the plantar region. As friction‐prone site, the heel lesions were rarely presented as Type 2. Subungual lesions primarily manifested as Type 2. The lesions on toes and fingers were more likely to evolve into Type 1.

### 3.3 Pathological characteristics

**FIGURE 2:**
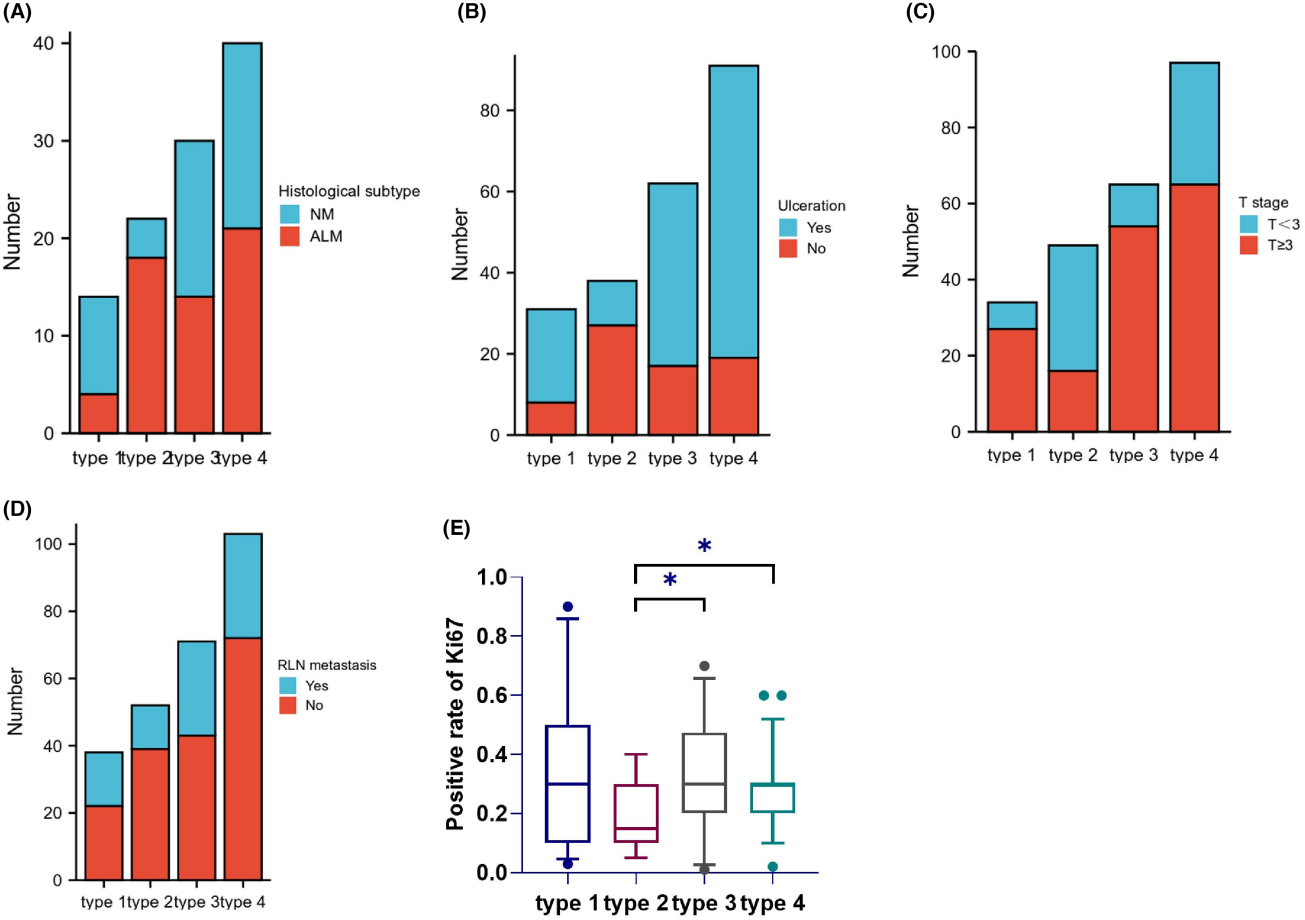
Pathological characteristics of the four types. (A) Common histological subtypes of the four types. (B) Ulceration rates of the four types. (C) T stages of the four types. (D) RLN metastasis of the four types. (E) The positive rate of Ki67 of the four types. RLN: regional lymph node.

A total of 136 patients had clear histological subtypes documented. The most prevalent subtypes were acral lentiginous melanoma (ALM) (41.9%) and nodular melanoma (NM) (36.0%). Notably, NM predominated in Type 1 (58.8%, 10 of 17), while ALM was more common in Type 2 (51.4%, 18 of 35) (Pearson's chi‐squared = 11.174, *p* = 0.011) (Table 1; Figure 2a; Table S3). The positive rate of Ki67 was lower in Type 2 compared to the other three types (*p* = 0.07, *p* = 0.03, *p* = 0.03) (shown in Figure 2e). Additionally, Type 2 had the lowest ulceration rate (28.9%, 11 of 38) among four types (shown in Figure 2b; Table S4). Type 2 also exhibited the earliest T stages (T < 3) in 67.3% of cases (33 of 49) (shown in Figure 2c; Table S5), along with the lowest rate of regional lymph node metastasis at initial diagnosis (25.0%) (shown in Figure 2d; Table S6). Conversely, Type 1 and Type 3 presented with more advanced T stages (T ≥ 3) in 79.4% (27 of 34) and 83.1% (54 of 65) of cases, respectively.

### 3.4 Genetic aberrations

**FIGURE 3:**
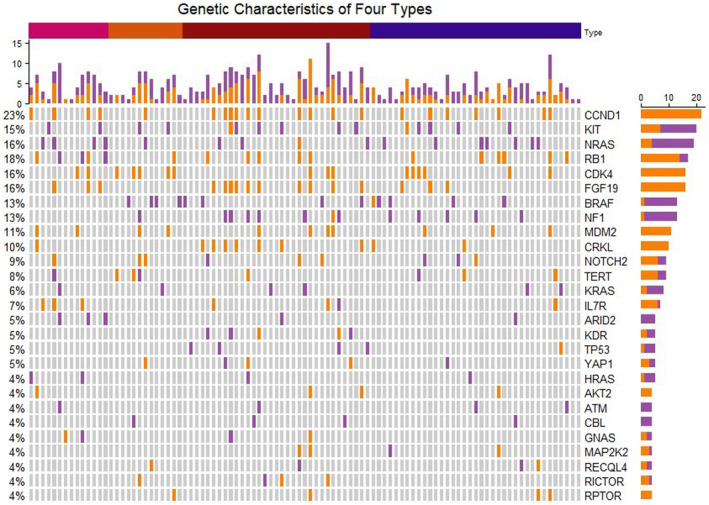
Common altered genes in NGS testing (*n* = 105). Orange blocks in the oncoprint mean copy number variations and purple blocks represent mutations. The four colors at the top of the oncoprint represent Type 1 to Type 4 in turn.

**FIGURE 4:**
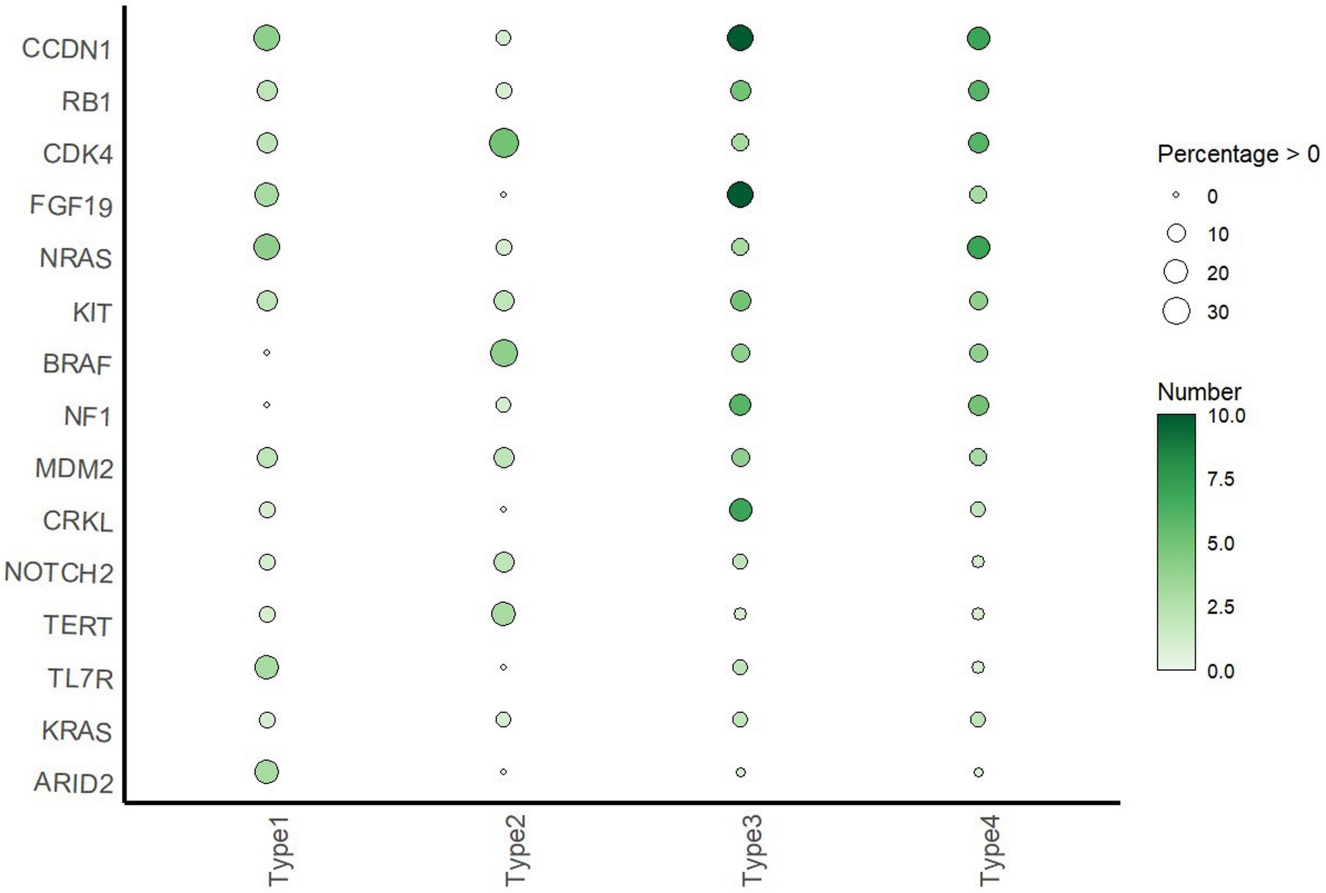
Bubble plot of top 15 genes. The horizontal coordinate indicates clinical classification, the vertical coordinate indicates the gene name, the depth of bubble color indicates gene alteration, and the bubble size indicates percentage of gene change in that type.

Out of 105 patients who underwent NGS testing, there were 15 Type 1 patients, 14 Type 2 patients, 37 Type 3 patients, and 39 Type 4 patients. The analysis revealed that copy number variations (CNVs) were more frequent in AM than somatic mutations based on NGS assay results (shown in Figure 3). Among these 105 patients, the top 10 altered genes (including somatic mutations and CNVs) were CCND1, KIT, NRAS, RB1, CDK4, FGF19, BRAF, NF1, MDM2, and CRKL. In Type 1 patients, the mutation rate of BRAF (0%, 0 of 15) was markedly lower than the overall rate, while the mutation rate of NRAS (26.7%, 4 of 15) was significantly higher. Conversely, Type 2 had the highest BRAF mutation rate (28.6%, 4 of 14), with the lowest NRAS mutation rate (7.1%, 1 of 14). KIT mutation rates remained relatively stable across all four types (ranging from 10.3% to 14.3%). In patients with a history of trauma at the primary site (Type 1 and Type 3), common genetic alterations included CNVs of CCND1, RB1, FGF19, IL7R, and ARID2 mutations, whereas CDK4 and TERT CNVs were less frequent. In contrast, Type 2 patients exhibited the lowest incidence of CCND1 and RB1 CNVs, but displayed the highest CDK4 CNV frequency. The variant frequencies of commonly altered genes were presented in Figure 4; Table S7 in the Supplement. AM typically exhibited low TMB levels and the median TMB across all types was 2.4mut/Mb, with Type 2 harboring the lowest TMB among the four types. However, there was no significant difference when comparing TMB among the four types (shown in Figure S2a) except when comparing TMB between patients with a trauma history (Type 1 and Type 3) and those without any traumatic factors (Type 2) (*p* = 0.021) (shown in Figure S2b).

### 3.5 Tumor immune microenvironment

**FIGURE 5:**
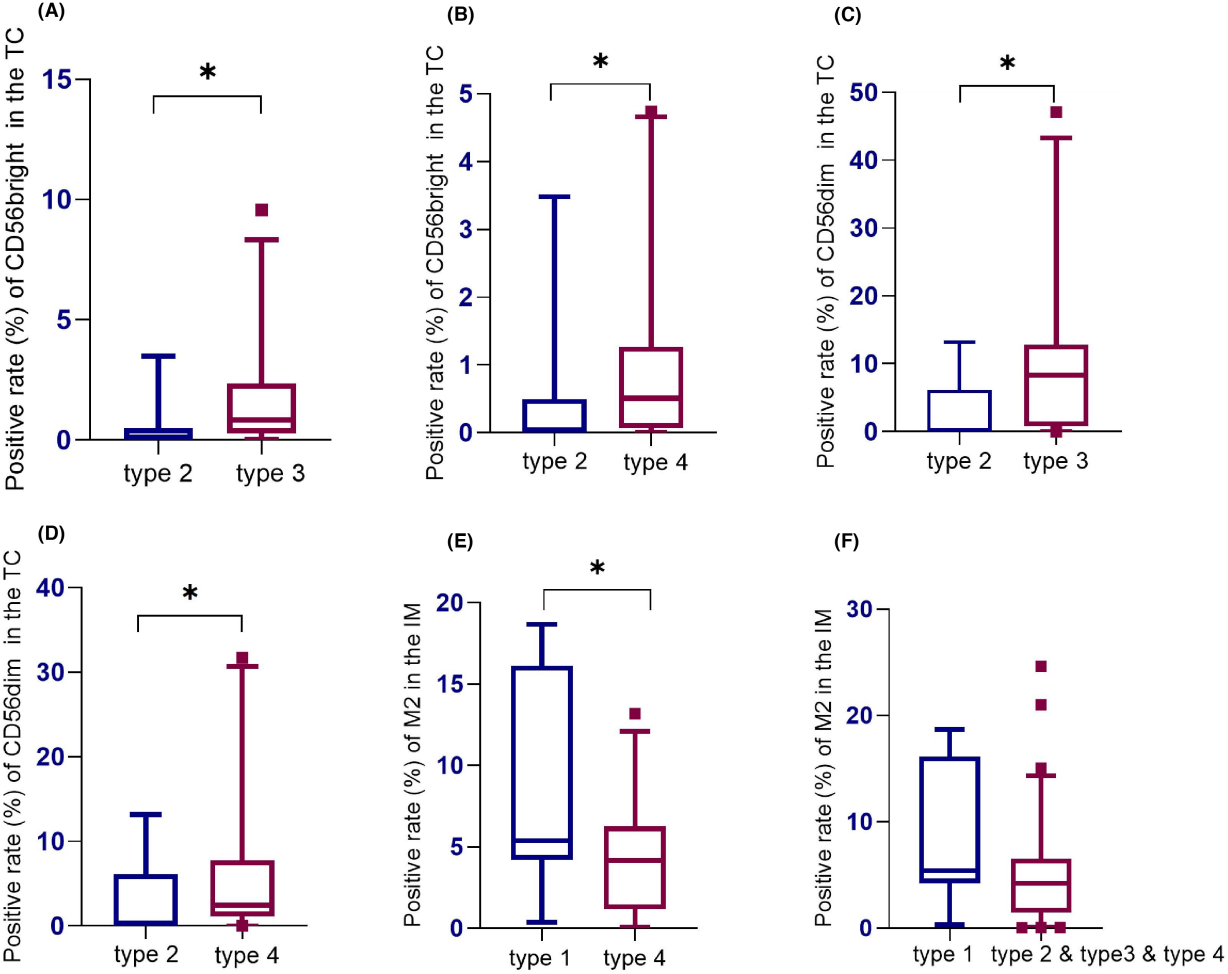
Differences in immune infiltration between different types. Two‐sample Mann–Whitney tests were performed and there were significant differences in CD56bright NK (A, B) and CD56dim NK (C, D) infiltration in the TC as well as M2 macrophages (E, F) in the IM. IM, invasive margin; TC, tumor center.

In general, AM displayed a low level of immune infiltration with in the microenvironment. Five types of immune cells (CD8^+^ T cell, M1 macrophage, M2 macrophage, CD56bright NK cell, and CD56dim NK cell) present in both the tumor center and invasive margin were subject to Kruskal–Wallis tests, but none of the results yielded statistical significance (shown in Figure S3). However, patients in Type 3 and Type 4 demonstrated significantly higher levels of CD56bright NK cell and CD56dim NK cell infiltration in the tumor center compared to Type 2 patients by two‐sample tests (*p* = 0.020, *p* = 0.039, *p* = 0.018, and *p* = 0.043, respectively) (shown in Figure 5a–d). Regarding M2 macrophages in the invasive margin, Type 1 patients exhibited significantly higher levels than Type 4 patients (*p* = 0.046), and patients with traumatic origin (Type 1) showed higher levels of M2 macrophages compared to patients with pigmented nevus origin (Type 2 and Type 3 and Type 4) (*p* = 0.066) (shown in Figure 5e,f). Additionally, there were no significant differences observed in the levels of other types of immune cells.

## 4 DISCUSSION

Our study aimed to investigate the proportion of different onset patterns in Chinese AM, with a specific focus on the role of trauma in AM. Our study showed that trauma accounted for a large proportion of AM in China, with Type1 and Type 3 accounting for 15.5% and 28.3%, respectively. Additionally, we conducted a comprehensive analysis of the clinicopathological features, genetic aberrations, and the tumor microenvironment among patients exhibiting varying onset characteristics, providing a possible microscopic rationale for the potential association between trauma and AM.

In comparison to other retrospective studies on AM,^12^, ^13^ our study observed a higher proportion of the NM subtype. BRAF mutation frequency and TMB were notably lower than those seen in non‐acral cutaneous melanoma (46%, 49.17mut/Mb).^14^ In our study, patients with traumatic histories exhibited higher TMB levels, possibly attributed to elevated cytokine levels and increased production of reactive oxygen species resulting from trauma and chronic inflammation, which may subsequently induce mutagenic DNA damage.^10^ CNVs were frequent in AM, in line with previous research.^15^ Furthermore, our study identified that CNVs in CCND1, RB1, FGF19, IL7R, and ARID2 mutations were more prevalent, while CNVs in CDK4 and TERT were less common in AM patients with a history of trauma. According to previous research, these commonly observed genetic aberrations in trauma‐associated melanoma have been reported to be related to microbial infections in a variety of infection‐related cancers.^16^, ^17^, ^18^, ^19^, ^20^, ^21^ For example, CCND1 amplification or cyclin D1 expression are associated with HPV infection in oral squamous cell carcinoma and cervical cancer.^16^, ^17^ FGF19 amplification is related to HBV or HCV infection and cirrhosis in HBV‐related hepatocellular carcinoma.^18^, ^19^ ARID2 expression can be inhibited by the X protein of HBV, an important viral protein in hepatocellular carcinogenesis, through weakening the binding of the transcription factor ATOH1 to the ARID2 promoter.^20^ Moreover, the HPV oncoprotein E7 inhibits RB1, thereby impairing its tumor suppressive function.^21^ Moreover, a recent study has shown the presence of microbiota in melanoma.^22^ It is therefore intriguing to explore whether a specific microbiota is present in trauma‐related melanoma and to investigate the role of microbes in oncogenesis. Understanding the interplay between trauma, genetic alterations, and infection in AM warrants further investigation. Such exploration may provide crucial insights into the complex etiology of AM in the Chinese population and potentially uncover novel avenues for prevention and treatment strategies.

Patients in Type 1 demonstrated the highest proportion of M2 macrophages in the invasive margin, indicating an immunosuppressive microenvironment. Conversely, Type 2 patients exhibited the lowest proportion of NK cells in the tumor center. In contrast, patients in Type 3 and Type 4 displayed a more favorable overall microenvironment. In a broader context, Type 2 presented the most favorable pathological and biological behavior, which was characterized by the lowest rate of the NM subtype, the significantly lowest Ki67‐positive rate, the highest ulcer‐free rate, the earliest T stages, and the lowest rate of lymph node metastasis. However, the proportion of NK cells of Type 2 patients in the tumor center was the lowest. Conversely, Type 1 primarily featured the NM histological subtype, advanced T stages, the highest NRAS mutation rate, and the highest M2 macrophage infiltration in the invasive margin. Nevertheless, the analysis of survival outcomes did not reveal significant differences among the four types, which may be attributed to limitations such as an insufficient sample size or the possibility that different clinical origins do not serve as decisive prognostic factors in AM. To date, there have been no reports of discernible prognostic variations between trauma‐associated melanoma and non‐trauma‐associated melanoma.

This study has several limitations. First, this is a single‐center retrospective study with a small sample size. Second, the unavailability of tumor tissues from some patients, particularly those who underwent surgery in other medical facilities, posed a constraint on our ability to conduct detailed examinations, such as histopathologic subtype assessments and genetic testing. Lastly, it is important to recognize the potential for recall bias in the medical histories provided by patients, which may have led to some degree of inaccuracy in the results. These limitations should be considered when interpreting the findings and underscore the need for further research with larger and more diverse cohorts to corroborate and extend our findings.

Despite the acknowledged limitations, our study represents a pioneering effort in characterizing the clinical classification of four distinct types within the Chinese AM population. Furthermore, it marks the first comprehensive investigation offering a potential microscopic basis for understanding the plausible relationship between trauma and AM. This study, which encompasses clinicopathological features, genetic aberrations, and tumor microenvironments, addresses a critical knowledge gap. Prior research on the association between trauma and AM has been predominantly limited to retrospective studies and case reports referring to clinical features, with a notable absence of exploration into the molecular level mechanisms. Our findings open up new avenues for further exploration and research in this domain.^5^, ^8^

Melanoma is a malignant cancer originating from melanocytes, which transforms from a single deteriorated melanocyte or a preexisting benign but dysfunctional nevus.^23^, ^24^ In this study, we refrained from definitively categorizing Types 2–4 patients as having preexisting nevi or de novo. We do highlight the repeated mention of traumatic events in AM patients. Our study underscores the importance of directing increased attention toward trauma‐onset AM in future clinical melanoma diagnosis and treatment, due to its distinctly aggressive biological nature. There is an imperative need to delve deeper into the mechanisms underpinning trauma‐associated melanoma, paving the way for more targeted interventions and therapeutic strategies. Furthermore, we emphasize the significance of science dissemination, particularly within the context of AM, and advocate for heightened efforts in educating the poorly educated segments of the population.

## 5 CONCLUSION

This study provides a novel clinical classification of Chinese AM based on diverse clinical onset characteristics and provides insight into the potential link between trauma and AM. Our study suggests that trauma‐related AM is common in China and presents the most unfavorable pathological and biological behavior. These findings highlight the importance and necessity of exploring trauma‐related AM. Further investigations are imperative to elucidate the underlying mechanisms governing the association between trauma and AM.

## AUTHOR CONTRIBUTIONS

**Rong Huang:** Conceptualization (equal); data curation (lead); formal analysis (lead); writing – original draft (equal); writing – review and editing (equal). **Mengke Zhao:** Data curation (supporting); formal analysis (supporting); investigation (equal); writing – original draft (equal); writing – review and editing (equal). **Guiying Zhang:** Data curation (supporting); investigation (supporting). **Yueling Yang:** Data curation (supporting); investigation (supporting). **Jiayu Wang:** Data curation (supporting); investigation (supporting). **Kelin Zheng:** Data curation (supporting); investigation (supporting). **Lin Li:** Methodology (supporting); visualization (supporting). **Xinyu Su:** Methodology (supporting); visualization (supporting). **Lianjun Zhao:** Conceptualization (supporting); resources (supporting). **Yirong Wu:** Data curation (supporting). **Zhengyun Zou:** Conceptualization (equal); funding acquisition (lead); project administration (lead); resources (lead); writing – review and editing (supporting).

## FUNDING INFORMATION

This work was supported by the National Natural Science Foundation of China (No. 82073365 and No. 81872484) and the Social Development Fund of Jiangsu Province (No. BE2019605).

## CONFLICT OF INTEREST STATEMENT

The authors have no conflicts of interest to declare.

## ETHICS STATEMENT

This study was approved by the Ethics Committee of the Affiliated Drum Tower Hospital, Medical School of Nanjing University (approval no. 2022‐170‐02).

## PATIENT CONSENT FOR PUBLICATION

Written or verbal informed consent was obtained from all patients.

## Supporting information


Figure S1.

Table S1.

## Data Availability

The data that support the findings of this study are not publicly available due to their containing information could compromise the privacy of research participants but are available from the corresponding author Zhengyun Zou.
