# Supplementary material for: Trauma plays an important role in acral melanoma: A retrospective study of 303 patients
Source: Cancer Med. 2024 Mar 28;13(7):e7137. doi: 10.1002/cam4.7137 (PMC10974717; doi:10.1002/cam4.7137)
Supplement: Supplementary file 1 — Figure S1. [file CAM4-13-e7137-s002.pdf]

Supplementary Material

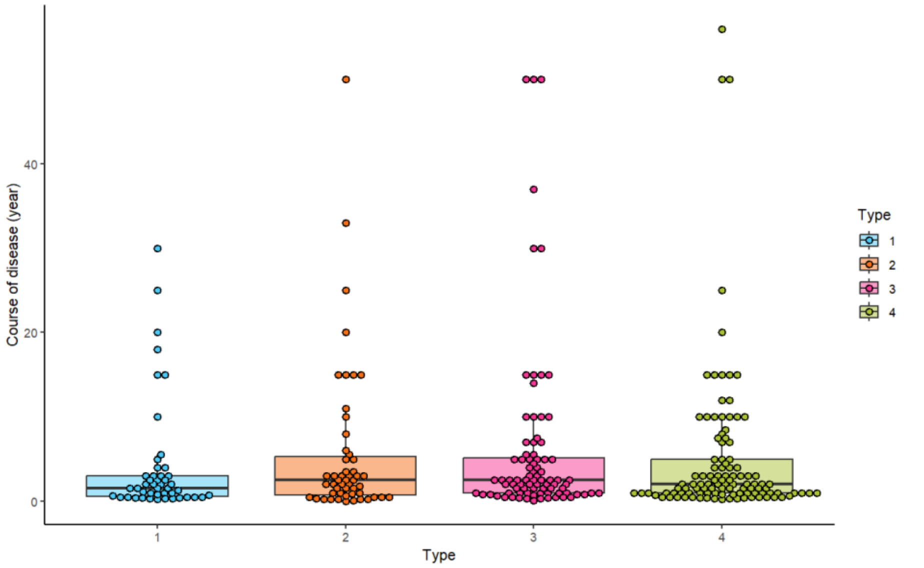

Fig. S1. The duration of lesion presence. There was no significant difference in the course of disease among the four groups (Kruskal Wallis Test, $P = 0.311$).

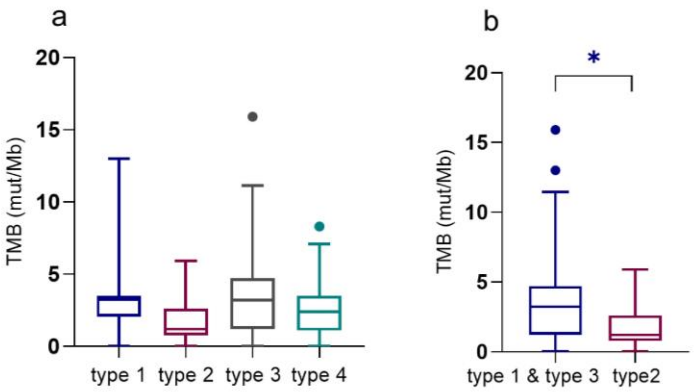

Fig. S2. TMB of the four types. There was no statistically significant difference in tumor mutation burden (TMB) among the four types. A significant difference was found when comparing TMB between patients with trauma history (type 1 and type 3) and those without traumatic events (type 2) ($P = 0.021$).

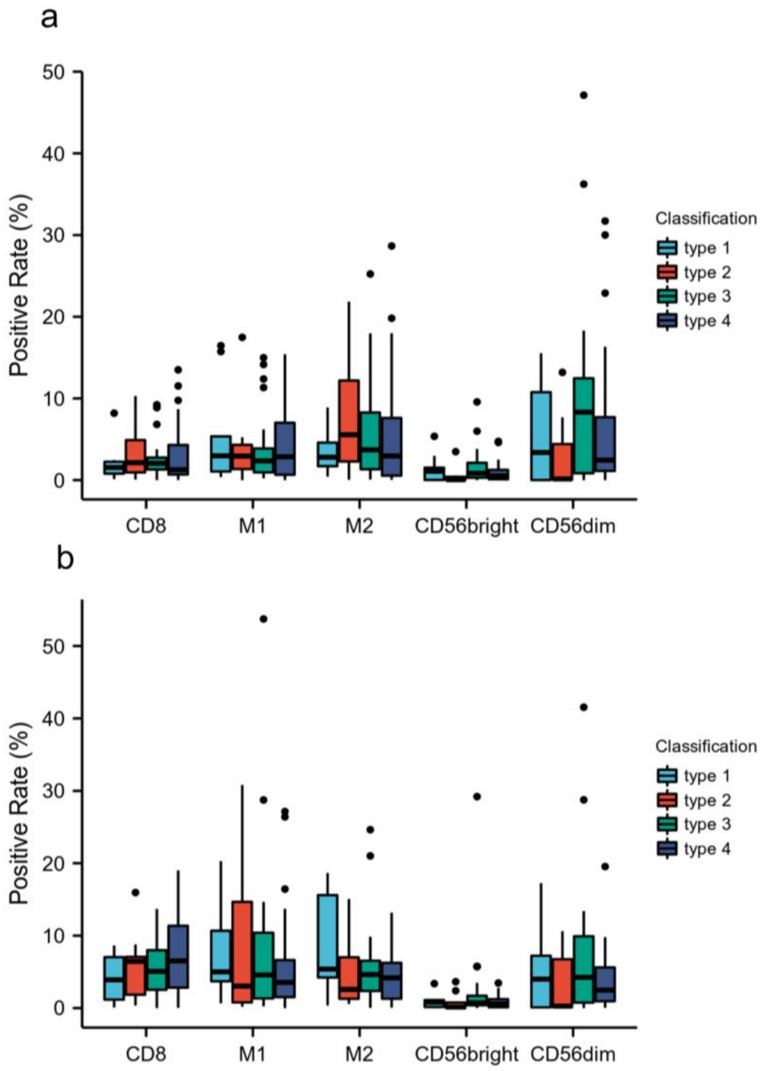

Fig. S3. The immune infiltration in the tumor center and invasive margin. Differences in immune cells ($CD8^+$ T cell, M1 macrophage, M2 macrophage, CD56bright NK cell, and CD56dim NK cell) in the tumor center (a) and invasive margin (b) among the four types were analyzed by Kruskal Wallis tests, and no significant results were shown ($P = 0.872$, $P = 0.954$, $P = 0.568$, $P = 0.104$, $P = 0.089$ in the tumor center, $P = 0.439$, $P = 0.732$, $P = 0.245$, $P = 0.530$, $P = 0.361$ in the invasive margin, respectively).
