# Supplementary material for: Trauma plays an important role in acral melanoma: A retrospective study of 303 patients
Source: Cancer Med. 2024 Mar 28;13(7):e7137. doi: 10.1002/cam4.7137 (PMC10974717; doi:10.1002/cam4.7137)
Supplement: Supplementary file 2 — Table S1. [file CAM4-13-e7137-s001.docx]

**Table S1. The median time of lesion presence among the four types.**

| Type | Type 1 | Type 2 | | Type 3 | Type 4 |
| --- | --- | --- | --- | --- | --- |
|  |  |  |  |  |  |
| Number | 46 | | 51 | 84 | 108 |
| Median  (Interquartile range)/year | 1.50 (2.75) | | 2.50 (4.90) | 2.50 (4.38) | 2.00 (4.00) |

**Table S2. The distribution of lesion sites.**

| Anatomical site | Left | Right | Total |
| --- | --- | --- | --- |
| Hands |  |  |  |
| Subungual | 15 | 10 | 25 |
| Palm | 1 | 0 | 1 |
| Fingers  Thumb  Index finger  Middle finger  Ring finger  Little finger | 9  4  1  3  0  1 | 11  5  2  2  2  0 | 20  9  3  5  2  1 |
| Feet |  |  |  |
| Subungual | 11 | 8 | 19 |
| Plantar | 61 | 79 | 140 |
| Heel | 26 | 29 | 55 |
| Toes  The big toe  The second toe  The third toe  The fourth toe  The little toe | 18  9  2  1  3  3 | 25  19  1  0  1  4 | 43  28  3  1  4  7 |

**Table S3. Constituent ratios of histological subtypes among the four types.**

| Histological subtypes | Type 1 | Type 2 | Type 3 | Type 4 | Total | chi-square test |
| --- | --- | --- | --- | --- | --- | --- |
|  |  |  |  |  |  | **χ^2^ *P* value** |
| NM | 10^a^ (71.4%) | 4^b^ (18.2%) | 16 (53.3%) | 19 (47.5%) | 49 (46.2%) | 11.2 0.011 |
| ALM | 4^a^ (28.6%) | 18^b^ (81.8%) | 14 (46.7%) | 21 (52.5%) | 57 (53.8%) |  |
| Total | 14 (100%) | 22 (100%) | 30 (100%) | 40 (100%) | 106 (100%) |  |

NM: nodular melanoma; ALM: acral lentiginous melanoma.

**Table S4. Ulceration rates of the four types.**

| Ulceration | Type 1 | Type 2 | Type 3 | Type 4 | Total | chi-square test |
| --- | --- | --- | --- | --- | --- | --- |
|  |  |  |  |  |  | **χ^2^ *P* value** |
| Yes | 23^a^ (74.2%) | 11^b^ (28.9%) | 45^a^ (72.6%) | 72^a^ (79.1%) | 151 (68.0%) | 33.0 <0.001 |
| No | 8^a^ (25.8%) | 27^b^ (71.1%) | 17^a^ (27.4%) | 19^a^ (20.9%) | 71(32.0%) |  |
| Total | 31 (100%) | 38 (100%) | 62 (100%) | 91  (100%) | 222 (100%) |  |

Different superscripts between columns indicate statistical difference (*P* < 0.05).

**Table S5. T stages of the four types.**

| T stage | Type 1 | Type 2 | Type 3 | Type 4 | Total | chi-square test |
| --- | --- | --- | --- | --- | --- | --- |
|  |  |  |  |  |  | **χ^2^ *P* value** |
| T<3 | 7^a^ (20.6%) | 33^b^ (67.3%) | 11^a^ (16.9%) | 32^a^ (33.0%) | 83 (33.9%) | 35.6 < 0.001 |
| T≥3 | 27^a^ (79.4%) | 16^b^ (32.7%) | 54^a^ (83.1%) | 65^a^ (67.0%) | 162 (66.1%) |  |
| Total | 34 (100%) | 49 (100%) | 65 (100%) | 97 (100%) | 245 (100%) |  |

Different superscripts between columns indicate statistical difference (*P* < 0.05).

**Table S6. RLN metastasis of the four types.**

| RLN metastasis | Type 1 | Type 2 | Type 3 | Type 4 | Total | chi-square test |
| --- | --- | --- | --- | --- | --- | --- |
|  |  |  |  |  |  | **χ^2^ *P* value** |
| Yes | 16 (42.1%) | 13 (25.0%) | 28 (39.4%) | 31  (30.1%) | 88 (33.3%) | 4.616 0.202 |
| No | 22 (57.9%) | 39 (75.0%) | 43 (60.6%) | 72  (69.9%) | 176 (66.7%) |  |
| Total | 38 (100%) | 52 (100%) | 71 (100%) | 103  (100%) | 264 (100%) |  |

RLN: regional lymph node.

**Table S7. The variant frequencies of commonly altered genes.**

| GENES | Type 1 | Type 2 | Type 3 | Type 4 |
| --- | --- | --- | --- | --- |
| CCND1 | 4（27%） | 1（6%） | 10（27%） | 7（18%） |
| RB1 | 2（13%） | 1（7%） | 5（14%） | 6（15%） |
| CDK4 | 2（13%） | 5（36%） | 3（8%） | 6（15%） |
| FGF19 | 3（20%） | 0 (0%) | 10（27%） | 3（8%） |
| NRAS | 4（27%） | 1（7%） | 3（8%） | 7（18%） |
| KIT | 2（13%） | 2（14%） | 5（14%） | 4（10%） |
| BRAF | 0 (0%) | 4（29%） | 4（11%） | 4（10%） |
| NF1 | 0 (0%) | 1（6%） | 6（16%） | 5（13%） |
| MDM2 | 2（13%） | 2（14%） | 4（11%） | 3（8%） |
| CRKL | 1（7%） | 0 (0%) | 7（19%） | 2（5%） |
| NOTCH2 | 1（7%） | 2（14%） | 2（5%） | 1（3%） |
| TERT | 1（7%） | 3（21%） | 1（3%） | 1（3%） |
| TL7R | 3（20%） | 0 (0%) | 2（5%） | 1（3%） |
| KRAS | 1（7%） | 1（6%） | 2（5%） | 2（5%） |
| ARID2 | 3（20%） | 0 (0%) | 1（3%） | 1（3%） |
